# Pregnancy-related changes in the canine serum N-glycosylation pattern studied by Rapifluor HILIC-UPLC-FLR-MS

**DOI:** 10.1038/s41598-024-71352-z

**Published:** 2024-09-06

**Authors:** Margareta Ramström, Martin Lavén, Ahmad Amini, Bodil Ström Holst

**Affiliations:** 1 Swedish Medical Products Agency, P. O. Box 26, 751 03 Uppsala, Sweden; 2 Department of Clinical Sciences, Swedish University of Agricultural Sciences, P. O. Box 7054, 750 07 Uppsala, Sweden

**Keywords:** Glycomics, Animal physiology

## Abstract

Canine reproduction differs from that of many other domestic animals, and increased knowledge on biochemical changes during canine pregnancy is important for investigations of infertility or subfertility. The total glycosylation pattern, i.e., the glycome, of body fluids reflects cellular status in health and disease. The aim of the present pilot study was to investigate pregnancy-related changes of the serum N-glycome in bitches. A method based on Rapifluor HILIC-UPLC-FLR-MS was optimized and applied for analysis and quantification of N-glycans in canine serum. Serum samples from six pregnant and five non-pregnant bitches, collected at four well-defined time points, were included. The levels of sialylated and galactosylated complex glycans were significantly elevated in serum from pregnant bitches, consistent with previous reports on human pregnancy. The levels of fucosylated and agalactosylated glycans decreased significantly in pregnant dogs. In non-pregnant dogs, the glycosylation pattern did not change during the cycle. Pregnancy is an inflammatory state, but our findings during canine pregnancy are quite the opposite to changes that have previously been described for dogs with a known parasitic infection. Evaluation of the canine glycome may thus be valuable in studies of canine pregnancy, possibly differing inflammatory changes related to pregnancy to those caused by an infection.

## Introduction

Dogs have several important functions in society, as beloved pets and family members, but also as assistance dogs, police dogs, military dogs, scent-detecting dogs and more. Because of this, dog breeders have an important role to produce healthy dogs of different breeds, suitable for the wide array of tasks. Bitches typically only experience two oestrous cycles per year^1^, resulting in a limited number of possible pregnancies from a single bitch. This is challenging for the breeders, and tools for diagnosing and possibly treating conditions with a negative impact on fertility are important.

Canine reproduction has some special features. The low number of oestrous cycles per year is one, another is the fact that the progesterone-producing, luteal, phase, is of a similar length irrespective if the bitch is pregnant or not^1^ –no luteolytic factor has been described in non-pregnant bitches. While progesterone concentrations are similar between pregnant and non-pregnant bitches, a few other factors differ between these groups. Relaxin is a hormone that is only produced by pregnant bitches^2^, and variations in the concentration of several inflammatory markers, including acute phase proteins (APPs) have also been reported^3–8^. Changes in serum protein levels during canine pregnancy have been investigated both by targeted^3–8^ and un-biased^9^ analytical approaches. The concentration of the APP fibrinogen A increases during pregnancy; a few studies show a significant increase around day 21–30^4,8,9^, while another investigation indicates an increase at week 5–6^7^. Significantly increased levels of C-reactive protein^3–5,8^, haptoglobin^6,7^ and ceruloplasmin^6,7^ have also been reported at various time-points during gestation. Changes in APP concentrations have been confirmed by a mass spectrometry-based proteomics study^9^ that also identified pregnancy-related changes related to the coagulation system. The clinical value of APPs as markers of pregnancy is limited since any inflammatory condition may cause elevated concentrations, and the markers are indeed non-specific. However, increased APP concentrations in well characterized and clinically healthy animals are an indication of pregnancy.

Not only protein concentrations, but also the extent and types of post-translational modifications may contribute with important information on biochemical events during canine pregnancy. Glycosylation is the most diverse and one of the most common post-translational modification of proteins. The majority of all mammalian proteins is estimated to be glycosylated^10,11^. Thorough investigations of individual proteins are frequently performed and review articles summarizing current knowledge on the glycosylation of the most abundant plasma and serum glycoproteins have been presented^11,12^. Characterization and routine testing of the glycosylation of biopharmaceuticals is a regulatory requirement, since it is well-established that even small changes in the glycosylation pattern may influence both efficiency and safety of a biological product^13–15^. Recent advances in chromatographic separation, mass spectrometry and automatization has enabled analysis of the glycome, i.e., the total repertoire of glycans, of cells, tissues and body fluids. Human serum glycosylation has been extensively studied and it has been demonstrated that the glycome reflects overall cellular status in health and disease^11,16,17^. Changes in glycosylation can e.g. modulate inflammatory responses^16^, and in pregnancy there is a need for modification of the immune system to ensure maternal-foetal tolerance. Pregnancy-associated changes in the serum N-glycome in women include changes in the sialylation and in the antennarity of the total serum N-glycome^18,19^.

Changes in the glycome pose an interesting alternative for pregnancy diagnosis, but, to date, there is limited knowledge on serum glycosylation profiles of other mammalian species than humans, and glycome changes related to pregnancy in the bitch have not been described. Few studies describe the canine serum glycome^20,21^. A thorough comparison between the canine and human serum N-glycome revealed differences in the levels of glycans and confirmed the presence of the two non-human epitopes, galactose-alpha-1,3-galactose (α-gal) and N-glycolylneuraminic acid (NGNA), in canine serum^21^. Furthermore, changes in canine serum related to progression of an infection with a parasite, *Dirofilaria immitis* (heartworm) have been investigated^20^. Galactosylation and core fucosylation increased, while sialylation decreased in infected dogs.

The aim of the present study was to investigate pregnancy-related changes of the serum N-glycome in bitches. To achieve this, an analytical method was developed and optimised for N-glycan quantification in canine serum. The study was designed as an exploratory pilot study involving a limited number of canine samples. Blood samples from six pregnant and five control bitches were collected at three well-defined time points, i.e., at the day of mating, in mid and in the late phase of pregnancy. Four dogs were also sampled at an earlier time point. Overall, our results indicate that changes in N-glycosylation occur during pregnancy and that these changes can be detected after 22 days of pregnancy.

## Results

### Glycan identification

**Fig. 1:**
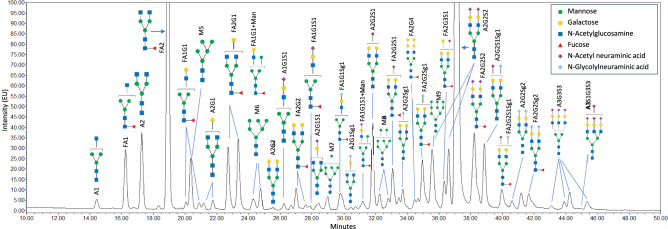
HILIC-FLR chromatogram of released and RapiFluor-labeled N-glycans from dog serum. Chromatogram zoomed, full scale chromatogram available in Fig. 1, Supplementary data.

A method based on RapiFluor-labelling and subsequent HILIC-UPLC-FLR-MS analysis ^22^ was developed and optimized for N-glycans in canine serum. A representative chromatogram is shown in Fig. 1. Structural assignment of glycan peaks was performed by mass spectrometry, tandem mass spectrometry and glycan reference standard analysis. In total, glycan structures were assigned to 50 peaks (Fig. 1, Supplementary Fig. S1, Supplementary Table S1–S4). The identified glycans were mainly complex diantennary structures and the two most abundant N-glycan species were A2G2S2 and FA2. The structural assignment and elution pattern were compared to and demonstrated to be in line with previously reported N-glycan analysis of dog serum^21^.

The glycans were categorized into different groups to allow for further evaluation. The selected groups were sialylated, fucosylated, high mannose, terminally galactosylated, galactosylated and agalactosylated complex glycans, and the categorisation of each glycan is described in Supplementary Table S1. Galactosylated glycans constituted around 75% of the total N-glycan content, and around 70% of the N-glycans were sialylated (refer to Table 1). Approximately 30% of the N-glycans were found to be fucosylated. The majority of the fucosylated glycans were confirmed to be core-fucosylated by MS/MS analysis and detection of the diagnostic ion at m/z = 679.7, analogous to the core fucose diagnostic peak previously reported for procainamide labeling^23^. Furthermore, 5% were high mannose structures, 18% were agalactosylated complex glycans and 13% were terminally galactosylated.

**Table 1:** Changes of serum N-glycosylation in pregnant and control dogs.

Glycan group	Category	Day 1 Abundance [min; max] (%)	Difference compared to Day 1 Average difference [min; max] (% points) and *p*-value		
			Day 22	Day 29	Day 43
Sialylated glycans	Pregnant (n = 6)	72.0 [63.6; 80.9]	3.2 [− 2.1; 10] NS	4.9 [1.7; 11] *p* = 0.028	5.2 [− 0.6; 12] *p* = 0.022
	Control (n = 5)	69.3 [63.7; 74.3]	− 1.5 [− 7.8; 1.6] NS	0.55 [− 3.1; 4.7] NS	− 0.13 [− 6.7; 3.8] NS
Fucosylated glycans	Pregnant (n = 6)	29.9 [23.4; 35.9]	− 3.9 [− 8.9; − 0.02] *p* = 0.038	− 4.8 [− 8.2; − 2.8] *p* = 0.010	− 4.3 [− 9.0; 1.3] *p* = 0.020
	Control (n = 5)	30.3 [27.5; 34.6]	0.49 [− 1.5; 5.0] NS	− 0.20 [− 3.4; 2.7] NS	− 0.30 [− 5.0; 4.7] NS
High mannose glycans	Pregnant (n = 6)	4.95 [3.8; 8.4]	0.02 [− 0.73; 0.79] NS	− 0.13 [− 1,6; 0,57] NS	− 0.25 [− 1.1; 0.40] NS
	Control (n = 5)	5.48 [4.56; 6.43]	0.57 [− 0.16; 1.4] NS	0.16 [− 0.20; 0.88] NS	0.65 [− 0.49; 2.13] NS
Terminally galactosylated glycans	Pregnant (n = 6)	13.7 [9.70; 21.6]	− 1.4 [− 2.5; − 0.88] NS	− 2.1 [− 5.0; − 0.76] NS	− 0.94 [− 4.8; 3.8] NS
	Control (n = 5)	12.5 [10.8; 15.7]	0.16 [− 0.58; 1.1] NS	0.16 [− 0.67; 1.1] NS	− 0.092 [− 2.5; 1.8] NS
Galactosylated glycans	Pregnant (n = 6)	75.9 [67.3; 81.8]	3.0 [− 1.2; 9.4] *p* = 0.070	3.9 [1.4; 9.1] *p* = 0.022	4.6 [1.8; 10] *p* = 0.008
	Control (n = 5)	74.5 [67.7; 79.9]	− 1.3 [− 6.7; 0.80] NS	0.47 [− 2.5; 4.0] NS	− 0.42 [− 5.8; 2.2] NS
Agalactosylated complex glycans	Pregnant (n = 6)	17.1 [11.7; 24.6]	− 3.0 [− 9.6; 1.1] NS	− 3.7 [− 7.2; − 1.4] *p* = 0.021	− 4.2 [− 8.7; − 1.7] *p* = 0.009
	Control (n = 5)	18.4 [12.6; 24.6]	0.54 [− 1.4; 5.1] NS	− 0.64 [− 3.7; 1.6] NS	− 0.73 [− 3.9; 3.3] NS

The differences in percentage points compared to the optimal day of mating (Day 1) are monitored for Days 22, 29 and 43 during pregnancy and the corresponding days for the control dogs. Significant changes (*p* < 0.05) were confirmed for sialylated, fucosylated, galactosylated and agalactosylated complex N-glycan, applying repeated measures ANOVA and post-hoc analysis by Dunnett’s correction for multiple comparison. NS denotes statistically non-significant changes.

### Pregnancy-related changes

**Fig. 2:**
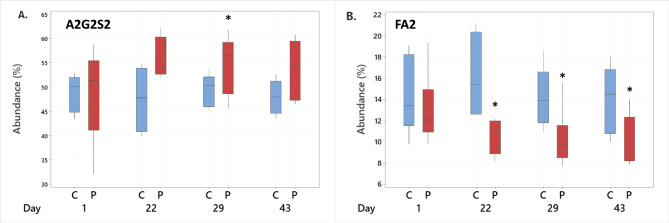
Abundance of the two dominating N-glycans in canine serum during pregnancy shown as % of the total N-glycan content. Box plots for control (C, blue boxes, n = 5) and pregnant bitches (P, red boxes, n = 6) sampled at the same days of the oestrous cycle are shown for glycan A2G2S2 (**A**) and glycan FA2 (**B**). Asterisks (*) indicate significant changes as compared to Day 1. For pregnant bitches, an increase of 6 percentage points was observed for A2G2S2 (*p* < 0.05 Day 29). A significant decrease of 3 percentage points was detected for FA2 for all three time points (*p* < 0.05). No changes over time were observed for control bitches.

**Fig. 3:**
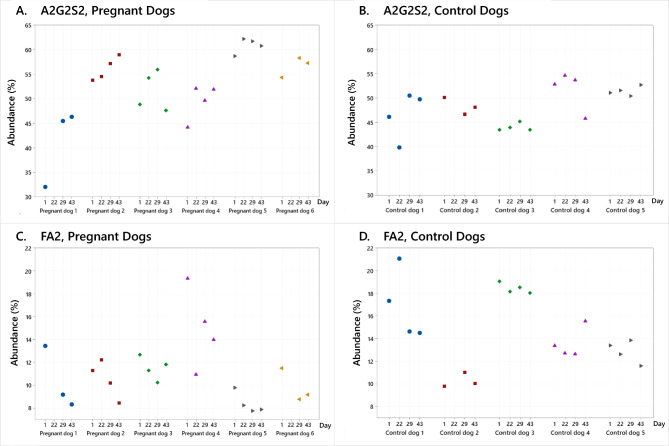
Abundance of N-glycans A2G2S2 and FA2 in serum of all individual dogs included in the study. (**A**) An increase in the level of A2G2S2 was observed for all pregnant dogs Day 22 and 29, and for all but one dog Day 43, as compared to Day 1. (**B**) No common trends were observed for control dogs. (**C**) A decrease in the level of FA2 was observed for all pregnant dogs Day 29 and 43, and for all but one dog Day 22, as compared to Day 1. (**D**) No common trends were observed for control dogs.

The serum N-glycome of eleven bitches was determined at three individual timepoints during the oestrous cycle, i.e., Day 1, Day 29 and Day 43. An extra timepoint, Day 22, was included for four pregnant and four non-pregnant dogs. The dominating glycan was for all dogs and all timepoints A2G2S2, constituting on average 49% of the total N-glycan content at Day 1 both for pregnant and control bitches. The second most abundant glycan was FA2, constituting on average 13% and 15%, respectively for the two groups. The abundances of these two glycans at the four timepoints for pregnant and control bitches are shown in Fig. 2. While the levels of both glycans remained stable over time for control bitches, the relative abundance of A2G2S2 increased by approximately 6 percentage points and the levels of FA2 decreased by 3 percentage points for pregnant bitches. The levels of A2G2S2 and FA2 for all individual dogs at the four timepoints are shown in Fig. 3, and the corresponding data is available in Supplementary Tables S5–S6. It is noted that the changes determined to be statistically significant (Fig. 2) were consistent for all individual pregnant dogs (Fig. 3). For A2G2S2, there was an increase as compared to Day 1 for all dogs and timepoints, except for one dog Day 43 (Fig. 3A). For FA2, a decrease as compared to Day 1 was detected for all dogs and timepoints, except for one dog Day 22 (Fig. 3C). No common trends were noted for control dogs (Fig. 3B and D).

Except for A2G2S2 and FA2, no other individual glycan constituted more than 3% of the total N-glycan content at Day 1 on average for all dogs. These glycans were therefore not evaluated individually, but in groups of related glycans. Changes over time were evaluated for sialylated, fucosylated, high mannose, terminally galactosylated, galactosylated and agalactosylated complex glycans. The results are summarised in Table 1 and numerical values for all dogs are included in Supplementary Tables S7–S12. Significant changes were confirmed for sialylated, fucosylated, galactosylated and agalactosylated complex N-glycan, applying repeated measures ANOVA and post-hoc analysis by Dunnett’s correction for multiple comparison. In fact, significant changes were observed both at Day 29 and Day 43 for all four glycan groups, increased abundance was noted for sialylated and galactosylated glycans, while there was a decrease for fucosylated and agalactosylated complex glycans. The alterations were also observed for these glycan groups at Day 22, even though the changes were solely found statistically significant (*p* < 0.05) for the fucosylated glycans at this time point. At Day 29, increased abundance of sialylated and galactosylated glycans and decreased levels of fucosylated and agalactosylated complex glycans were confirmed for all six pregnant dogs, as shown by the min–max range. No significant changes in the levels of any of the glycan groups were noted for control bitches at the corresponding days in the oestrous cycle. Supplementary Table S13 shows the HILIC-FLR chromatograms for the Day 1 and Day 29 serum samples for all 11 dogs, and the abundances of all identified N-glycans are tabulated.

### Method performance and optimization

The RapiFluor-HILIC-UPLC-FLR-MS method was optimized with respect to sample preparation and chromatography. A target volume equivalent to 0.75 µL serum was selected and the robustness of the method was studied by varying the serum volume. Additionally, during previous glycan analysis in our laboratory the importance of buffer exchange prior to N-glycan deglycosylation and labelling was noted^24^, whereby this aspect was additionally studied. It was concluded that the method provided consistent results over the studied range, that the selected target volume was appropriate, and that filtration of samples was not needed (Supplementary Fig. S2).

The chromatographic separation was optimized by increasing the length of the gradient and by increasing the concentration of ammonium formate in the mobile phase. The latter gave a significant improvement of the peak shape of late eluting, sialylated glycans, presumably by mitigating detrimental ionic secondary interactions (Supplementary Fig. S3). A higher ammonium formate concentration could however lead to decreased MS signals, due to ion suppression, but the improved chromatography significantly counteracted any such effects, with a two-fold increase of peak height for A2G2S2 (200 mM vs 50 mM ammonium formate, Supplementary Fig. S4).

The variability of the optimized method was evaluated by repeated sample preparation and analysis of an assay control sample, i.e., a serum sample from a non-pregnant bitch. The sample was prepared at 10 different occasions using four individual RapiFluor-MS N-Glycan Kits, referred to as Kit 1–4. The resulting intermediate precision data are presented in Table 2. A higher precision was obtained for samples prepared within a kit, compared to the overall precision attained from all four kits. Additionally, repeatability precision was obtained by repeated injection of the assay control sample (Supplementary Table S14). From this data it was concluded that sample preparation introduced most of the variability of the obtained results, but that the overall precision still was considered acceptably low.

**Table 2:** Intermediate precision data obtained from sample preparation and analysis of the assay control sample, performed on 10 different occasions, using 4 different sample preparation kits (data from kit 4, n = 1, not separately displayed). Abundance (%) and standard deviation (SD) are presented.

Glycan group/Glycan	Kit 1—4 Abundance (SD) % (n = 10)	Kit 1 Abundance (SD) % (n = 3)	Kit 2 Abundance (SD) % (n = 3)	Kit 3 Abundance (SD) % (n = 3)
Sialylated	71.0 (4.8)	74.0 (4.1)	65.2 (0.7)	72.4 (3.0)
Fucosylated	26.2 (2.6)	25.3 (2.0)	29.4 (0.3)	24.7 (1.8)
High mannose	5.6 (0.7)	4.9 (0.4)	6.2 (0.0)	5.9 (0.4)
Terminally galactosylated	13.3 (0.8)	12.8 (0.4)	14.3 (0.1)	13.1 (0.6)
Galactosylated	75.4 (3.8)	77.9 (3.1)	70.7 (0.5)	76.2 (2.3)
Agalactosylated complex glycans	16.6 (3.2)	14.8 (2.9)	20.6 (0.6)	15.4 (1.9)
A2G2S2	49.8 (3.6)	51.6 (2.2)	45.2 (0.3)	51.2 (2.5)
FA2	11.2 (2.1)	10.1 (1.9)	13.8 (0.4)	10.3 (0.9)

## Discussion

We here describe, for the first time, pregnancy-related changes in the total serum glycosylation pattern for the dog. The levels of sialylated glycans were elevated during canine pregnancy, which is in line with what has been confirmed for human pregnancy^19,25^. Few studies have been conducted on other species, but increased sialylation has also been reported for the camel^26^. The increase for dogs was observed already at Day 22 and a significant increase of around 5 percentage points was detected on Day 29, remaining during Day 43. In addition, the total proportion of galactosylated glycans was elevated Day 29, similar to the situation in human pregnancy, in which the levels of IgG galactosylation have been demonstrated to increase^27,28^. In contrast, the levels of fucosylated and agalactosylated glycans decreased Day 29. This is similar to the situation in the camel, in which a decrease of fucosylated glycans has been described^26^. It should be noted that pregnancy-associated changes are not straight-forward to compare between species since the overall glycan pattern differs between mammals^21,29^ and since the pregnancy length and placenta types are different. However, the findings of the present study further support that at least some glycosylation events are similar between species.

While the increase in sialylation and galactosylation during pregnancy generally agrees with what has been observed for other species, the results are opposite to what has been reported for dogs infected with *Dirofilaria immitis*^20^. Changes in the overall N-glycan pattern can be caused both by changes in the relative abundances of glycoproteins in serum and by modification of the glycan structures, i.e. the glycosylation microheterogeneity, of one or several glycoproteins. Many of the APPs, including alpha-1-acid glycoprotein, haptoglobin and alpha-1-antitrypsin, are glycoproteins. Altered levels of individual APPs have been reported during canine pregnancy^6,7,9,30^. In addition, an increase in glycoprotein acetyls (GlycA), as measured by NMR, has been observed during canine mid-pregnancy^31,32^. The levels of GlycA can be regarded as a combined response for several glycoprotein APPs, and GlycA has been suggested an inflammatory biomarker for systemic inflammation^33^. It is thus well-established that changes related to abundant serum glycoproteins occur in mid-pregnancy. However, our study is the first to monitor the changes at the level of individual glycans and glycan groups. In dogs infected by *Dirofilaria immitis*, the decrease in sialylation was considered most likely related to changes in the overall glycoprotein pattern, in particular an increase in the IgG level, resulting in higher relative abundance of FA2 but lower abundance of A2G2S2^20^. The changes in N-glycan pattern observed in our study could be due to changes in other glycoprotein levels, as compared to the dogs infected by *Dirofilaria immitis,* but it is also likely that they are related to changes in glycan microheterogeneity of abundant serum proteins, including APPs.

The method chosen for N-glycomics analysis in the present study, RapiFluor-HILIC-UPLC-FLR-MS, was initially developed for analysis of individual, purified glycoproteins^22^, but high throughput applications on human plasma^34,35^ have demonstrated good repeatability and robustness in more complex samples. To the best of our knowledge, this is the first application of RapiFluor-labeling of glycoproteins in canine serum. The sample consumption of the method developed in our laboratory is very low, around one µL of serum is sufficient for the analysis. This volume is minor as compared to the volume of blood samples collected from dogs; typically measured in mL. Thus, glycan analysis can be performed on serum samples extracted for other diagnostic purposes, which is in line with the 3Rs: Replace, Reduce, Refine. In addition, results from another study indicate that the N-glycan profile of canine serum remains stable during long-term storage at -80 °C for at least 25 years^20^. Thus, investigations of changes in the glycosylation pattern would be possible also for rare conditions where few samples can be collected over time. In the current study, samples were collected over a rather short total time period of 1.5 years and for each individual dog, blood samples were obtained during 42 days.

The method variability was studied by repeated sample preparation of an assay control, followed by HILIC-UPLC-FLR-MS analysis (Table 2). The variability within a sample preparation kit was lower than the total variability, encompassing four different kits. To avoid unwanted variability due to sample preparation, samples from individual dogs were therefore always prepared within a kit. The repeatability of the LC-method was of such a degree that highly robust retention times were obtained which facilitated identification of glycans in serum samples when using glycan reference standards (Supplementary Table S14). Additionally, the repeatability precision of the LC-method with respect to glycan abundance was high enough to enable single injection of samples, thereby avoiding time consuming replicate injections (Supplementary Table S14).

For healthy humans, the serum N-glycan profile has been demonstrated to be remarkably stable for individuals, but relatively variable within the population^17^. Our results confirm that the serum N-glycome is stable for non-pregnant bitches during the oestrous cycle. No significant changes were observed for any of the N-glycan groups, and the average difference in abundance as compared to Day 1 were for most glycan groups less than one percentage point (Table 1). Analogous to the situation in humans, the variability between dogs was demonstrated to be larger and the repeated measures design of the study was therefore considered to be advantageous. The significant changes observed for pregnant bitches were 5 percentage points for sialylated and fucosylated glycans and around 4% for galactosylated and agalactosylated complex glycans. Due to the variability observed between dogs it is suggested that if the N-glycan pattern, in future, will be used for diagnostic purposes, serum samples from the bitches should also be collected before mating to establish reference levels. It should be noted that the trends established to be statistically significant were confirmed for all six individual dogs at Day 29, i.e., there was an increase in the abundance of sialylated and galactosylated glycans and a decrease in fucosylated and agalactosylated glycans, even though the magnitude of the changes differed between dogs.

From a diagnostic point of view, the results obtained on N-glycosylation profile in this study are interesting. The changes observed in the glycan pattern, including altered levels of the individual glycans A2G2S2 and FA2, increased sialylation and galactosylation and decreased fucosylation, occurred already at D22, at the same time as we have previously described changes in the APPs fibrinogen and CRP^8,9^. However, the glycan pattern for pregnant dogs differs from that previously described for dogs with a known infection with *D. immitis*^20^, although infection with *D. immitis* has been described to cause an acute phase response with associated changes in the APPs^36^. This suggests that although changes in the glycan pattern were detected at the corresponding times to previously described changes in APP concentrations, the glycan pattern may not be caused by inflammatory changes or that there is a change in microheterogeneity of the APPs that differs between pregnancy and other inflammatory states. From a diagnostic point of view this is beneficial, as this means that the glycan pattern possibly is a more specific marker for pregnancy than the concentration of APPs, as the latter will change because of any inflammatory cause. However, the changes in glycan pattern related to bacterial or viral infections should also be investigated. The findings of this exploratory study should also be confirmed in an extended study including more dogs.

## Methods

### Materials

GlycoWorks RapiFluor-MS N-Glycan Kit, RapiFluor-MS Glycan Performance Test Standard, RapiFluor-MS High Mannose Test Standard and RapiFluor-MS Sialylated Glycan Performance Test Standard were purchased from Waters (Milford, MA, USA). Ammonium formate, eluent additive for LC–MS, LiChropur, ≥ 99.0%, was obtained from Supelco. Formic acid 99% for LC–MS, used for mobile phase preparations, formic acid, puriss. p.a., ACS reagent, reag. Ph. Eur., 98%, used for SPE clean-up and acetonitrile, HiPerSolv CHROMANORM for HPLC LC–MS grade—suitable for UPLC/UHPLC were purchased from VWR Chemicals.

### Dogs

Stored samples from eleven clinically healthy bitches of different breeds; six pregnant bitches (one each of Bernese mountain dog, Chow Chow, German shepherd, Golden retriever, Labrador retriever and Polish Lowland Sheepdog) and five non-pregnant control bitches (2 beagles, 1 each of mixed breed, Rottweiler and German shepherd), were included in the quantitative study. All pregnant bitches had delivered puppies. Samples from the bitches have previously been used for another study^8^. In addition, an assay control sample was used for system suitability studies, a healthy non-pregnant bitch (golden retriever).

### Sampling

Samples had been collected during the same period of the oestrous cycle from both groups. The first sample had been collected at the optimal mating time: a fully cornified vaginal smear and progesterone concentrations above 30 nmol/L (Day 1) and the following samples had been collected on days 22, 29 and 43. In total 44 blood samples had been collected from the cephalic vein into test tubes without additive.

### Sample preparation

Serum samples were initially diluted 1:10 in water in a 1.5 mL plastic tube, followed by mixing. The samples were thereafter prepared as described in the GlycoWorks RapiFluor-MS *N*-Glycan Kit (Waters, Milford, MA, USA); 7.5 µL of the diluted serum sample were added to 15.3 µL of water in a 1.5 mL plastic tube. 6 µL of a buffered solution containing 5% (w/v) RapiGest SF were added, followed by aspiration and dispensing to mix. The mixture was heat denatured for 3 min at a temperature exceeding 90 °C, using a heat block. The plastic tube was thereafter removed from the heat block to cool for 3 min at room temperature. 1.2 µL of Rapid PNGase F were added, followed by aspiration and dispensing for mixing. The mixture was thereafter incubated at 50 °C for 5 min, using a heat block. The tube was removed from the heat block to cool for 3 min at room temperature. 12 µL of a RapiFluor-MS reagent solution were added to the tube, followed by aspiration and dispensing for mixing. The ensuing labeling reaction was allowed to proceed for 5 min at room temperature, followed by addition of 358 µL of acetonitrile. The sample was thereafter cleaned using solid phase extraction (SPE), using the provided µElution plate and a Positive Pressure-96 Processor (Waters). Wells were initially conditioned with 2 × 200 µL of water, followed by equilibration with 200 µL of a solution of water/acetonitrile (15:85, v/v). The serum sample was thereafter loaded onto the well, followed by washing with 2 × 600 µL of a solution of formic acid/water/acetonitrile (1:9:90, v/v/v). Glycans were finally eluted with 3 × 30 µL of Glycoworks SPE elution buffer (200 mM ammonium acetate in 5% acetonitrile). The eluate was diluted with 310 µL of Glycoworks sample diluent-DMF/ACN and mixed. Diluted eluates were stored at − 80 °C, prior to HILIC-UPLC-FLR-MS analysis.

### HILIC-UPLC-FLR-MS analysis

A Waters Acquity LC-system was used with an Acquity FLR detector and an Acquity TQD MS detector (Waters). Acquisition and chromatographic data processing was performed using the software Empower (Empower 3, Waters). Mobile phase A consisted of 200 mM ammonium formate, adjusted to pH 4.4, using formic acid, and mobile phase B of acetonitrile. The column used was Acquity BEH Glycan Amide, 2.1 × 150 mm, 1.7 µm particles, operated at 60 °C. The gradient was set up as follows: initial flow rate at 0.4 mL/min, using a linear gradient from 0 – 60 min, with a change of mobile phase B of 75–54%. From 60 to 61.5 min the flow was changed from 0.4 to 0.2 mL/min and mobile phase B from 54 to 0%, keeping this setting at 61.5–64.5 min. At 64.5–68.1 min, B was changed from 0 to 75% and kept at this setting. At 68.1–72.6 min the flow was increased to 0.4 mL/min. 72.6–80 min the settings were as follows: 75% B and 0.4 mL/min. The autosampler temperature was set at 10 °C and an injection volume of 30 µL was used. The FLR detector was set as follows; excitation wavelength: 265 nm, emission wavelength 425 nm and data rate: 2 points/sec. The MS instrument was operated in ESI+ , scanning m/z 650–1365 at a scan rate of 1500 Da/sec, using a capillary voltage of 3 kV, cone voltage 20 V and an extractor voltage of 3 V. The source temperature was set at 120 °C and the desolvation temperature at 400 °C. The desolvation gas flow was operated at 600 L/hr, cone gas flow at 0 L/hr and collision gas flow at 0.1 mL/min, employing helium as collision gas.

### Glycan identification and quantification

Identification of glycans was performed by HILIC-UPLC-FLR-MS and glycan reference standard analysis. Obtained glycan mass values were compared with theoretical mass values and with mass values obtained from glycan reference standard analysis (Supplementary Table S1–S4). Obtained retention times were compared with those acquired from glycan reference standard analysis. Additional structural confirmation was achieved by controlled in-source fragmentation (MS/MS). Diagnostic ions were monitored to confirm the presence of N-Acetylneuraminic acid (NANA) (m/z 657.6), N-glycolylneuraminic acid (NGNA) (m/z 673.6) and core fucose (m/z 679.7).

The HILIC-UPLC-FLR-MS instrumentation enabled direct coupling of MS-data to the FLR-data, which facilitated correct assignment of FLR-peaks. Quantitative data, used for N-glycan calculations and comparisons, were generated from FLR chromatograms, using Area% of glycan peaks, as extracted by the chromatography software, i.e. Abundance (%). A reporting threshold of 0.05% was applied.

### Statistical analysis

Statistical analysis was performed in Minitab©. The glycan/glycan group levels were evaluated using repeated measures ANOVA for pregnant (n = 6) and non-pregnant (n = 5) dogs, respectively, at four timepoints; Day 1, Day 22, Day 29 and Day 43. Post-hoc analysis was performed applying the Dunnett’s correction for multiple comparison, setting Day 1 as the reference day. *P*-values < 0.05 were considered statistically significant.

### Ethical considerations

The study was approved by the Uppsala Ethical Committee of Animal Experimentation (C23/9) and the Swedish Board of Agriculture (31-1365/09). All experiments were performed in accordance with relevant guidelines and regulations. The dog owners gave their informed written consent. The study was performed according to these approvals and to the ARRIVE guidelines. The methodology described and the conclusions drawn in this paper are the authors’ opinion and not necessarily the standpoint of the Swedish Medical Products Agency.

## Supplementary Information

Supplementary Information.

Supplementary Table S13.

Supplementary Tables S1–S4.

Supplementary Tables S5–S12.

## Data Availability

Data generated or analysed during this study are for most parts included in this published article (and its Supplementary Information files). All datasets generated during and/or analysed during the current study are available from the corresponding author on reasonable request.
