# Supplementary material for: Pregnancy-related changes in the canine serum N-glycosylation pattern studied by Rapifluor HILIC-UPLC-FLR-MS
Source: Sci Rep. 2024 Sep 6;14:20861. doi: 10.1038/s41598-024-71352-z (PMC11379866; doi:10.1038/s41598-024-71352-z)
Supplement: Supplementary file 1 — Supplementary Information. [file 41598_2024_71352_MOESM1_ESM.docx]

# Supplementary information for

**Pregnancy-related changes in the canine serum N-glycosylation pattern studied by Rapifluor HILIC-UPLC-FLR-MS**

Margareta Ramström^1^, Martin Lavén^1^, Ahmad Amini^1^ and Bodil Ström Holst^2*^

^1^ Swedish Medical Products Agency, P. O. Box 26, SE-751 03 Uppsala, Sweden

^2^ Department of Clinical Sciences, P. O. Box 7054, Swedish University of Agricultural Sciences, 750 07, Uppsala, Sweden

* Corresponding author: Bodil Ström Holst, bodil.strom-holst@slu.se

**
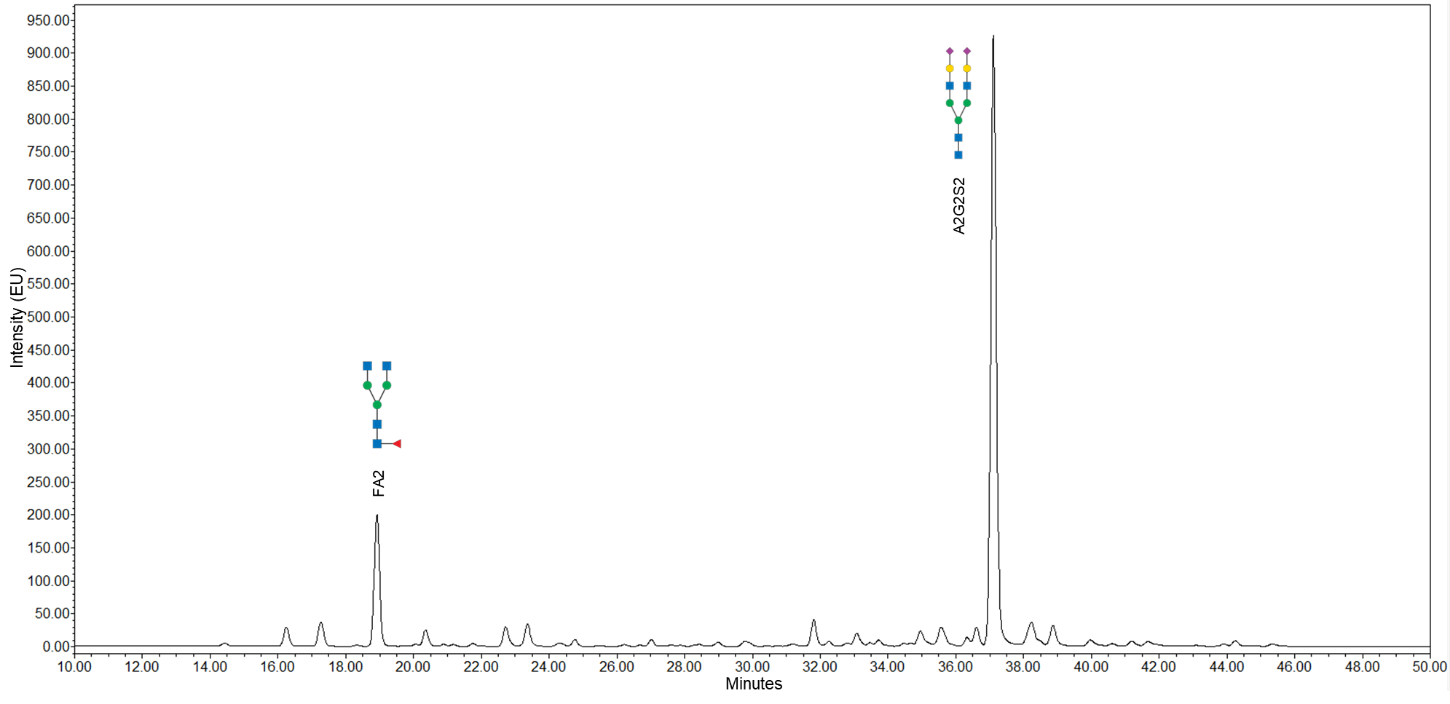
**

**Figure S1.** HILIC-FLR chromatogram of released and RapiFluor-labeled N-glycans from dog serum.

**Figure S2.** Variation of volume serum used for sample preparation (volumes of 0.375, 0.75 and 1.5 µL) and filtration (with or without), plotted against abundance (%) of glycans A2, FA2 and A2G2S2.


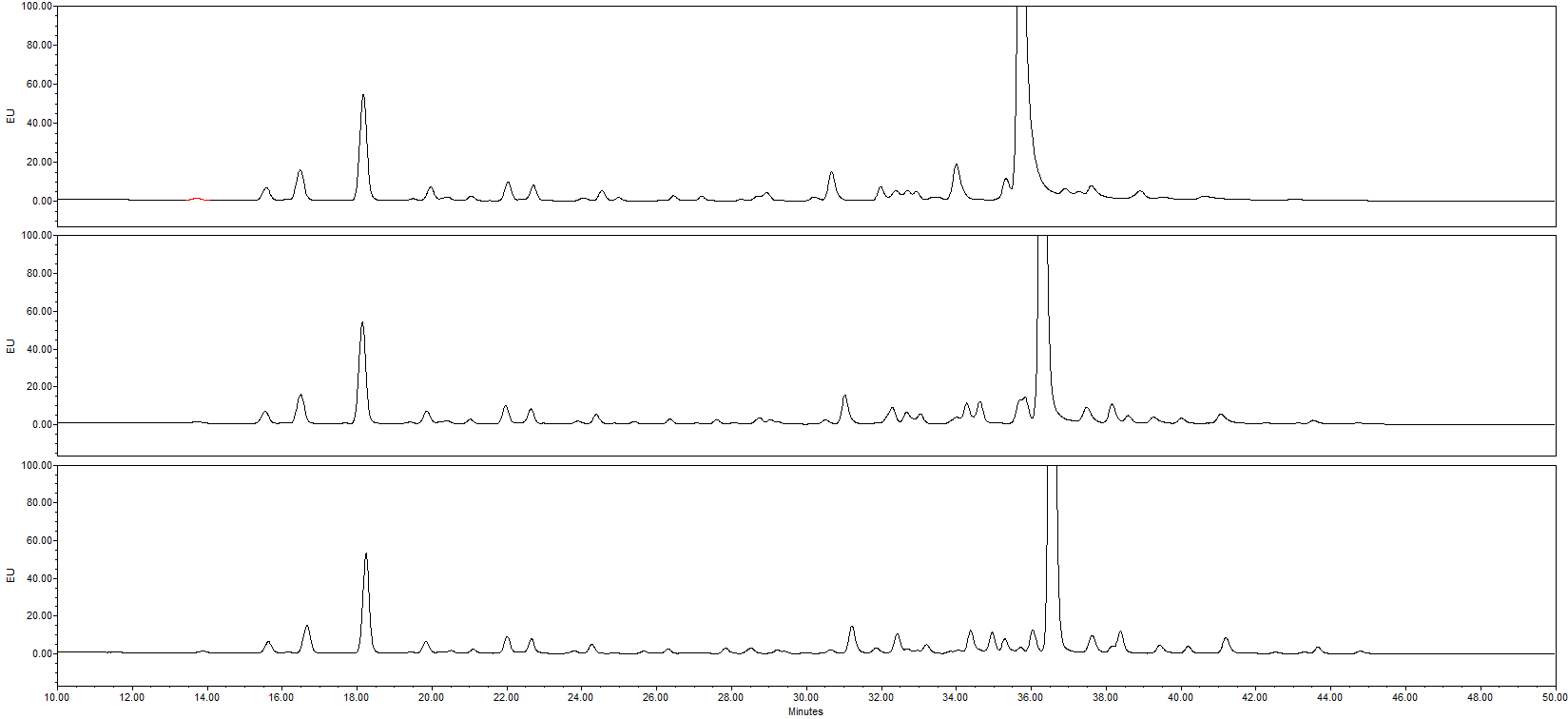


**Figure S3.** Chromatograms (HILIC-FLR) resulting from analysis using different concentrations of ammonium formate in the mobile phase. Top pane: 50 mM, middle pane: 100 mM and bottom pane: 200 mM ammonium formate. Symmetry factor A2G2S2; 1.75 (50 mM), 1.2 (100 mM) and 1.1 (200 mM).


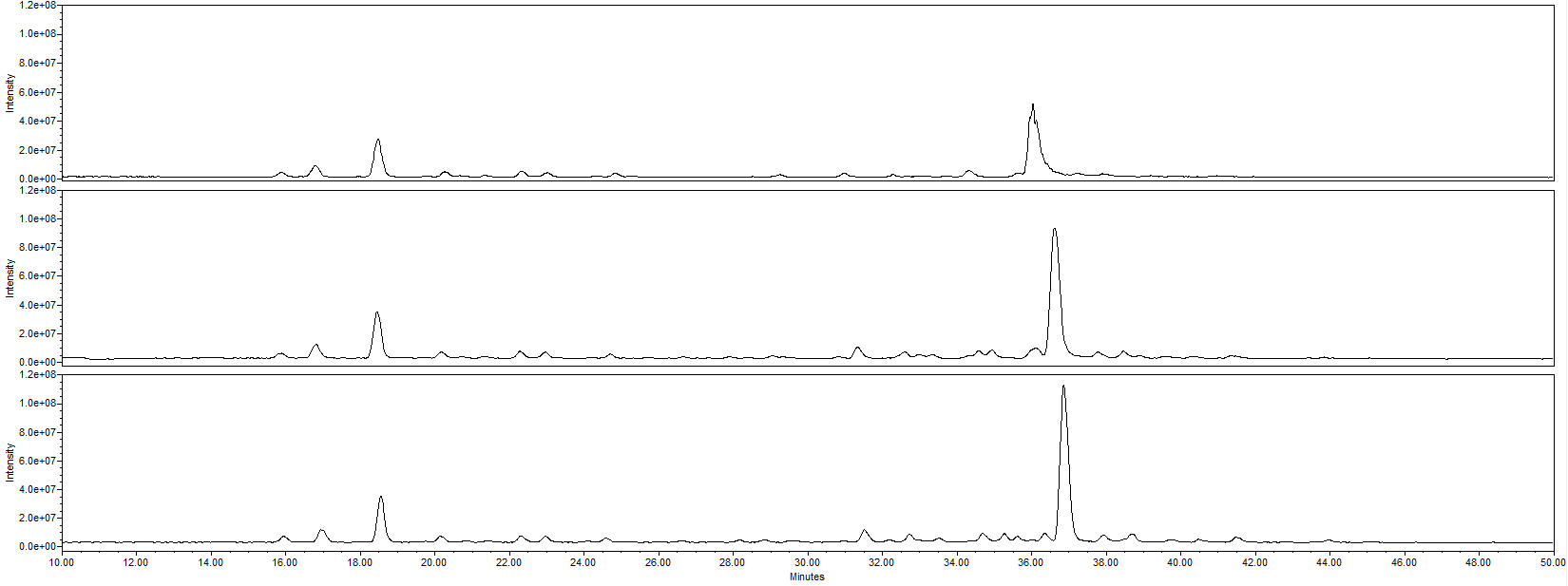


**Figure S4.** Chromatograms (HILIC-MS) resulting from analysis using different concentrations of ammonium formate in the mobile phase. Top pane: 50 mM, middle pane: 100 mM and bottom pane: 200 mM ammonium formate.

**Tables S1-S4.** **Proposed structures of RapiFluor-labeled N-glycans from dog serum.** (Refer to separate file, Supplementary Table S1-S4.xlsx)

**Tables S5-S12. Abundance of glycans and glycan groups as determined by Rapifluor- HILIC-FLR.** (Refer to separate file, Supplementary Table S5-S12.xlsx)

**Table S13. HILIC-FLR chromatograms and abundance of all N-glycans for the Day 1 and Day 29 serum samples for all pregnant and control dogs** (Refer to separate file, Supplementary Table S13.xlsx)

**Table S14.** Repeatability precision data obtained from repeated injection (n=3) of the assay control sample, performed on 8 different occasions. Standard deviation (SD) given for abundance and retention time.

|  | **Abundance** | | **Retention time** | |
| --- | --- | --- | --- | --- |
| **Occasion** | **A2G2S2**  **SD (%)** | **FA2**  **SD (%)** | **A2G2S2**  **SD (min)** | **FA2**  **SD (min)** |
| 1 | 0.11 | 0.06 | 0.080 | 0.064 |
| 2 | 0.04 | 0.05 | 0.074 | 0.058 |
| 3 | 0.28 | 0.14 | 0.061 | 0.045 |
| 4 | 0.29 | 0.09 | 0.011 | 0.011 |
| 5 | 0.32 | 0.12 | 0.013 | 0.010 |
| 6 | 0.14 | 0.08 | 0.005 | 0.005 |
| 7* | 0.30 | 0.01 | 0.032 | 0.021 |
| 8 | 0.04 | 0.03 | 0.002 | 0.013 |

*n=2.
